# Enzymatic degradation of maize shoots: monitoring of chemical and physical changes reveals different saccharification behaviors

**DOI:** 10.1186/s13068-020-01854-1

**Published:** 2021-01-05

**Authors:** Cécile Barron, Marie-Françoise Devaux, Loïc Foucat, Xavier Falourd, Rachelle Looten, Maud Joseph-Aime, Sylvie Durand, Estelle Bonnin, Catherine Lapierre, Luc Saulnier, Xavier Rouau, Fabienne Guillon

**Affiliations:** 1 CIRAD, INRAE, IATE, Institut Agro, Univ. Montpellier, 34060 Montpellier, France; 2 INRAE, UR BIA, 44316 Nantes, France; 3 INRAE, BIBS Facility, 44316 Nantes, France; 4 Institut Jean-Pierre Bourgin, INRAE, AgroParisTech, Université Paris-Saclay, 78000 Versailles, France

**Keywords:** Lignocellulosic, Plant dry fractionation, Recalcitrance, Particles size, Image analysis, Time-lapse study, Porosity, Biomass saccharification

## Abstract

**Background:**

The recalcitrance of lignocellulosics to enzymatic saccharification has been related to many factors, including the tissue and molecular heterogeneity of the plant particles. The role of tissue heterogeneity generally assessed from plant sections is not easy to study on a large scale. In the present work, dry fractionation of ground maize shoot was performed to obtain particle fractions enriched in a specific tissue. The degradation profiles of the fractions were compared considering physical changes in addition to chemical conversion.

**Results:**

Coarse, medium and fine fractions were produced using a dry process followed by an electrostatic separation. The physical and chemical characteristics of the fractions varied, suggesting enrichment in tissue from leaves, pith or rind. The fractions were subjected to enzymatic hydrolysis in a torus reactor designed for real-time monitoring of the number and size of the particles. Saccharification efficiency was monitored by analyzing the sugar release at different times. The lowest and highest saccharification yields were measured in the coarse and fine fractions, respectively, and these yields paralleled the reduction in the size and number of particles. The behavior of the positively- and negatively-charged particles of medium-size fractions was contrasted. Although the amount of sugar release was similar, the changes in particle size and number differed during enzymatic degradation. The reduction in the number of particles proceeded faster than that of particle size, suggesting that degradable particles were degraded to the point of disappearance with no significant erosion or fragmentation. Considering all fractions, the saccharification yield was positively correlated with the amount of water associated with [5–15 nm] pore size range at 67% moisture content while the reduction in the number of particles was inversely correlated with the amount of lignin.

**Conclusion:**

Real-time monitoring of sugar release and changes in the number and size of the particles clearly evidenced different degradation patterns for fractions of maize shoot that could be related to tissue heterogeneity in the plant. The biorefinery process could benefit from the addition of a sorting stage to optimise the flow of biomass materials and take better advantage of the heterogeneity of the biomass.

## Background

The development of a plant biorefinery for the treatment of lignocellulosic biomass to produce biomolecules, bioenergy and biomaterials in substitution to fossil carbon is a major challenge today [1]. The biological conversion of cell wall polysaccharides is more attractive than other types of industrial conversion [2] because it offers the possibility to produce specific products with low energy input in an ecofriendly environment. However, with no pretreatment, the conversion yield is low. Many factors are responsible for the recalcitrance linked to both biomass and enzymes [3]. Lignin content is the main component involved in recalcitrance [4, 5]. Other components more specific to grass cell walls, *p*-coumaric acid (CA) and ferulic acid (FA), have been widely investigated in the context of the saccharification performance of grass lignocellulosics [6–9]. Hemicelluloses, and to a lesser extent pectins, are thought to limit cellulose accessibility thereby hindering efficient enzymatic hydrolysis [10–13]. On the contrary, it has been also suggested that hemicelluloses through their substituents could affect positively the biomass enzymatic digestibility by reducing cellulose crystallinity [14–16]. Cellulose structure, especially crystallinity, has been also linked to saccharification but results have been conflicting [17, 18] or reported little or no effect [19, 20]. At the scale of polymers, the degree of polymerization of the cellulose [14, 21–23], presence of acetyl groups [24] as well as the occurrence of sugar substituents can also limit enzymatic hydrolysis by preventing enzymes from accessing glycosidic linkages reviewed by [25]. Many pretreatments before the saccharification step aim to reduce the detrimental impact of these components [12, 14, 26–32].

In addition to the influence of the chemical composition of the biomass and of the chemical structure of the constituent polymers, the role of particle size and porosity that determine the surface accessible to enzymes has already been investigated [14, 19, 33–36]. Conflicting effects of a decrease in particle size on the conversion rates have been reported depending on the substrate, the resulting particle size range or the percentage of fine particles [34, 37]. Several authors, using either solute exclusion [19, 36, 38–40] or Simons’ staining [14, 20, 41, 42] techniques, reported positive relationships between the internal surface area or pore surface accessible to cellulase and cellulose enzymatic hydrolysis yield and rate. Several methods to investigate the biomass-water interaction have been implemented to explain the recalcitrance phenomenon, including water retention value and time-domain low field NMR (LF-NMR). Water retention measures the capacity of the biomass to retain water against a centrifugal force. It has been used as an indirect measure of cell wall properties including overall hydrophilicity and porosity [43, 44] and has been shown to be correlated with saccharification yield [35, 45–47]. LF-NMR relaxometry provides more detailed information on the physical and chemical environment of water in the biomass [33, 35, 45, 47–49]. This approach revealed that the presence of constrained water favored hydrolysis [45, 49].

To summarize, no real consensus has been reached on the most important causal factors aside from cellulose accessibility and the detrimental role of lignin content. The heterogeneity of the tissues forming the lignocellulosic biomass is also known to cause differences in saccharification patterns [50]. Several authors have investigated the behavior of different cell types in enzymatic degradation through imaging [8, 51–53] and tissue extraction [54–56]. They demonstrated differences in tissue susceptibility that varied with the nature of the cell walls. Lignocellulosic particles subjected to saccharification originate from different tissues. Anatomical constituents or even tissues could be obtained through dry fractionation [57, 58], which combines grinding and separation processes. Powder enriched in specific anatomical parts of plant is affordable and the initial complexity in bulk samples due to the plant heterogeneity could thus be reduced.

In the present work, it is proposed to separate biomass particles into fractions that differ in their physical state and composition and analysed separately their saccharification pattern. By this way, we aimed at a better understanding of the phenomena of recalcitrance. Maize was chosen as a feedstock since it has several characteristics that are suitable for bioconversion. It is mainly grown for the grain so the stalk biomass can be used for biofuel production. Dry fractionation was applied to obtain the fractions [59]. In addition to the chemical composition of the unfractionated maize shoot and derived fractions, the present work focused on the physical–chemical properties of particles: original particle size distribution, specific surface area, and porosity. NMR techniques were used to evaluate particle porosity, cellulose accessibility and biomass-water interactions at different water contents [45, 48]. A torus reactor prototype designed for image acquisition during the reactions was used for the real-time monitoring of the size and number of particles to identify the effect of physical changes in the particles resulting from the release of sugars during degradation. We hypothesized that measuring both physical and biochemical parameters in contrasting populations of particles would help decipher the mechanisms of the enzymatic reaction.

## Results and discussion

### Differences in particle reactivity during saccharification assays of maize shoot powder

**Fig. 1:**
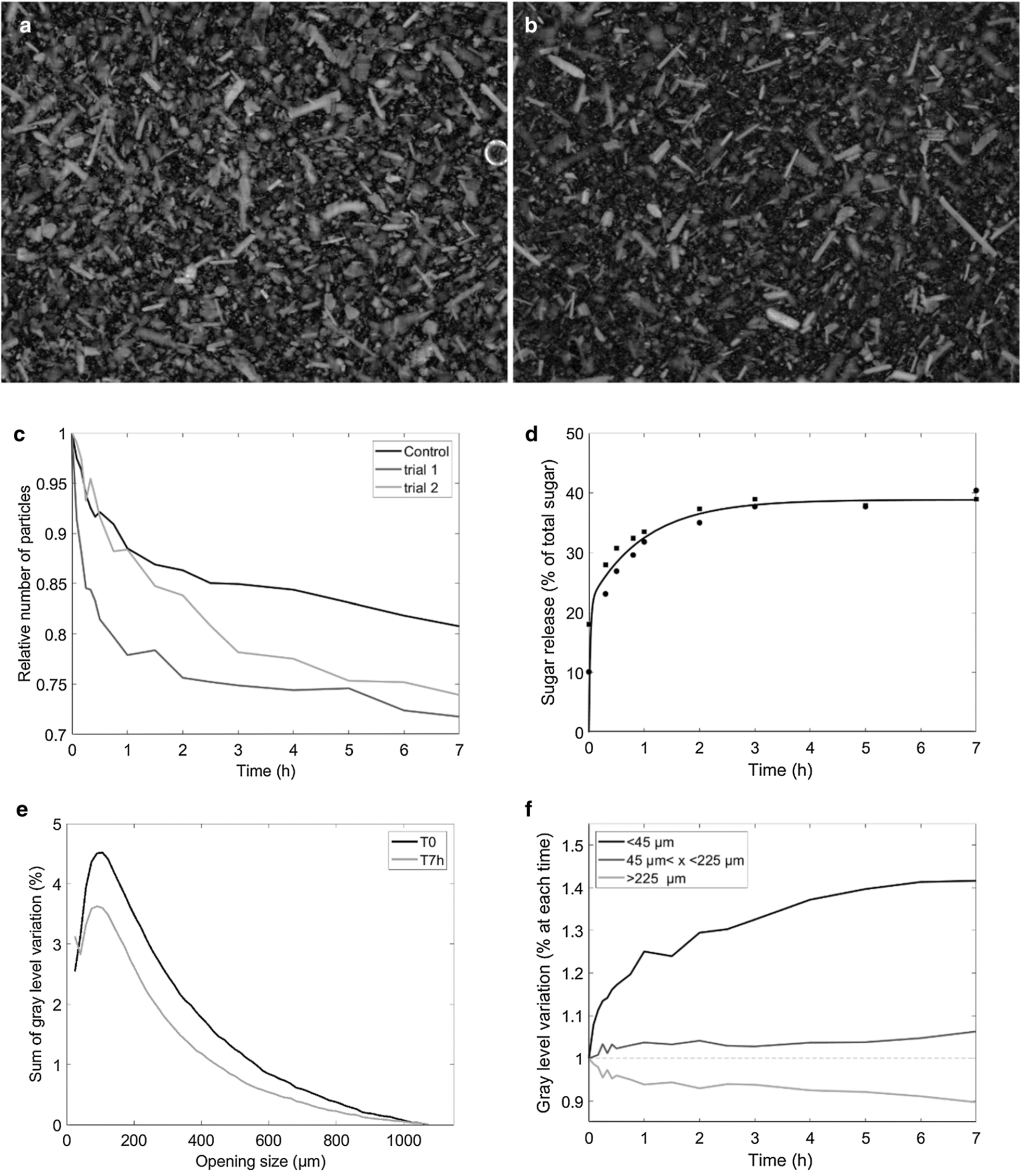
Saccharification of maize powder in the torus reactor Cinetore. **a** Picture of the original sample **b** Picture of the sample after 7 h of saccharification. Field of view: 10 × 13.3 mm^2^. **c** Changes in the relative number of particles measured as the sum of gray levels relative to time T0 in the control experiment and two enzymatic degradation trials. **d** Percentage of sugar released according to hydrolysis time. **e** Granulometric curves of the original sample and after 7 h saccharification. M–T0: time T0 normalized to 1, M–T7 h: time 7 h normalized to 1, M–7 h norm T0: time 7 h normalized relative to the amount of particles at time T0. f) Changes in the three classes of particles relative to their original number

First a saccharification assay of the unfractionated maize shoot powder (M) was carried out using the torus reactor Cinetore to analyze the saccharification yield coupled with real-time monitoring of changes in the number and size of the particles through image analysis. Before degradation, the maize shoot powder was made of particles of different sizes and shapes (Fig. 1a), including long rod-shaped particles. The longest particles were as long as 1,500 µm, (measured by hand).

In the control experiment, the stirring into the reactor caused the disappearance of 19% of the particles and the release of 2.1% of total sugars. This result suggests that some particles were broken down by the mechanical action of the screw and/or the water. After seven hours of saccharification, the number of particles observed was lower (Fig. 1b). Changes in the total sum of gray levels relative to time T0 were computed (Fig. 1c) to account for the number of particles in the reactor during saccharification. The number of particles rapidly decreased to about 75% after one hour. After 7 hours, 26–28% of the particles disappeared. In parallel, the amount of sugars released was evaluated from the hydrolysates (Fig. 1d). The percentage of disappearance rapidly increased at first and then stabilized at 36–37% after 2 hours. This result is consistent with results obtained in non-pretreated maize [44]. Clearly, some particles were recalcitrant to enzymatic hydrolysis while others disappeared or were modified.

Changes in particle size during the reaction were also studied. Figure 1e shows the granulometric curves at times T0 and 7 h. The granulometric curves normalized relative to time T0 showed variations both in the number of particles and in the decrease in size. A mode was observed at 90 µm for time T0 and 75 µm for time 7 h. Three size classes were built from these granulometric curves: [0–45 µm], [45–225 µm], [> 225 µm]. The three classes represented, respectively, 6%, 44% and 50% of the original sample at T0, and 8%, 47% and 45% of the sample after 7 h of hydrolysis. Changes in the number of particles in each class were evaluated after normalization relative to their original number (Fig. 1f). A strong relative increase in small particles from 1 to 1.40 and a slight relative increase in medium particles from 1 to 1.06 were observed. In parallel, the number of large particles decreased slightly from 1 to 0.90.

Three interpretations are possible. The first, a certain proportion of large, medium, and small particles were fully degraded while others were recalcitrant. The full degradation for most of the particles can be ruled out as most of the tissues in maize shoot are lignified [60, 61]. The second, some medium and large particles were degraded and broke into many small particles thereby increasing the relative proportion of small particles. The third, medium and large particles may be partly eroded during degradation or broken down into medium size particles. Whatever the size, some particles were recalcitrant as small, medium, and long particles were still present after seven hours of degradation. To better understand this heterogeneous behavior, maize shoot powder was sub-fractioned to isolate fractions that were expected to be more homogeneous according to the saccharification process.

### Exploring variability of maize shoot powder

**Fig. 2:**
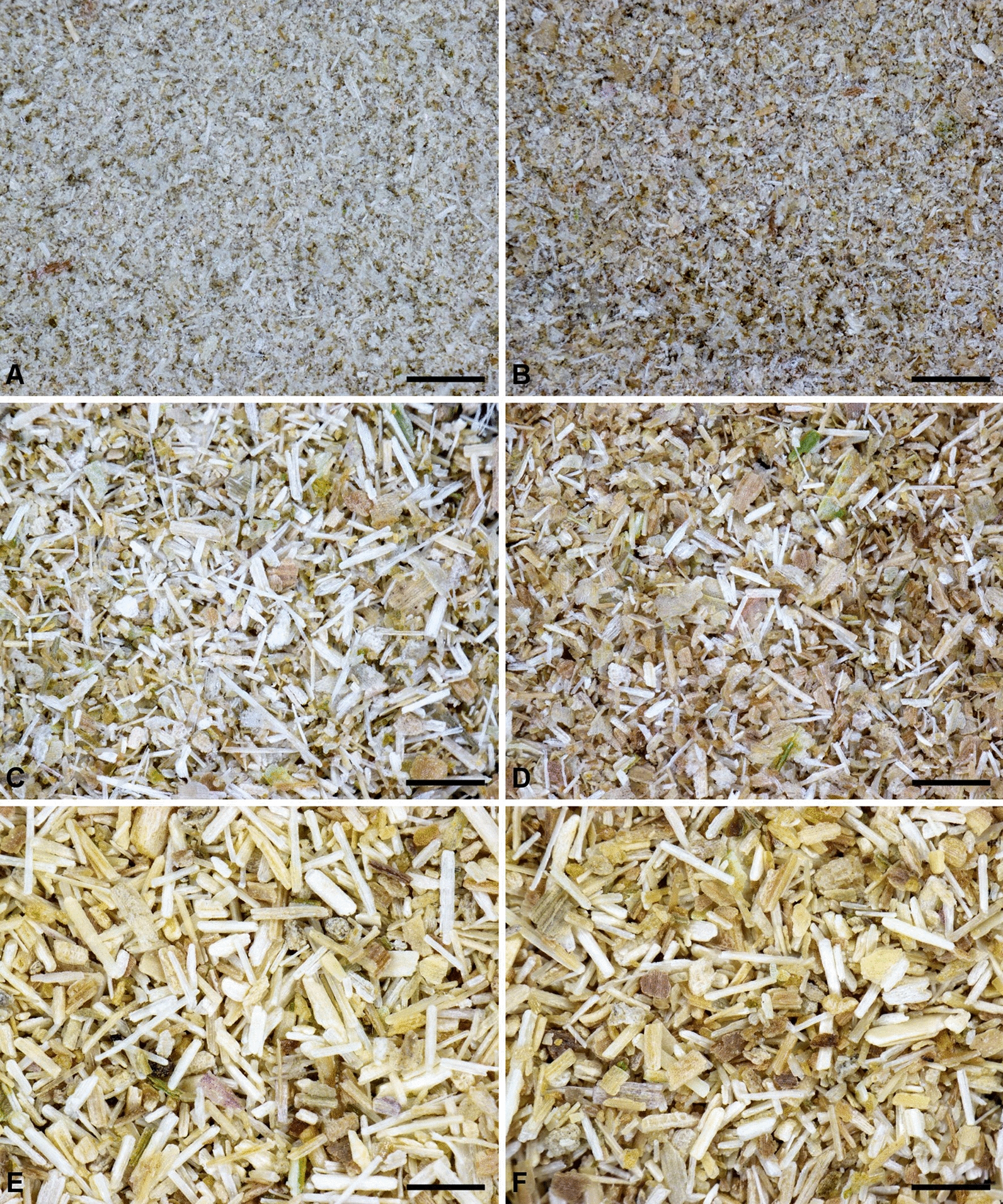
Optical observation of fractions of maize shoot powder: **a** M*f* + , **b** M*f*-, **c** M*m* + , **d** M*m*-, **e** M*c* + , **f** M*c*- (the bar scale represents 500 µm)

The enzymatic hydrolysis of each particle might be linked to the origin of the tissue in the maize shoot. To explore this hypothesis, dry fractionation was carried out to try to obtain fractions with specific tissue enrichment from leaves, pith, or rind. The particle size obtained in the fractions is not only the result of the efficiency of the grinder but is also due to interactions between plant structures and mechanical loading modes developed in the grinder [59]. The particle size could thus reflect differences in grinding ability in relation to the mechanical properties of the tissues. On this basis, we chose a separation process mainly based on particle size characteristics [62, 63]. Air classification enabled the separation of the original maize shoot powder into three fractions (fine, medium, and coarse). A subsequent step was undertaken to take advantage of differences in particle composition [64]. Electrostatic separation, based on tribocharging, which is an interface phenomenon that depends on surface composition [65], was then used to separate each fraction into two more fractions, giving a final total of six fractions (Fig. 2). The coarse, medium, and fine fractions accounted for, respectively, 43%, 22% and 35% of the weight of the original sample (Table 1). Their median particle sizes (D_50_) were 310–315 µm, 159–201 µm, and 58–63 µm, for the coarse, medium, and fine fractions, respectively (Table 1). Size dispersion was still observed within each fraction but was less than in the unfractionated maize shoot sample, as shown by the narrower range of span values. Electrostatic separation led to no further substantial separation according to particle size, as shown by optical observations (Fig. 2) and by laser granulometry (Table 1). The median particle size of samples recovered on the negative electrode side (coded “−”) was slightly higher than on the positive ones (coded “ + ”), in the coarse, medium, and fine fractions. As expected, the reduction in particle size was consistent with an increase in a specific surface area measured by physisorption (Table 1). Moreover, the hue of the particles separated by electrostatic separation also differed from the negative fractions with more brownish hues than in the positive fractions.

**Table 1:** Yield of maize shoot fractions and physical characteristics of maize shoot powder and its fractions

Sample	Yield (% dm)	D_50_(µm)	Span	Ssp (m^2^.g^−1^)
M		171.3	2.9	0.97
M*c*-	18.5	315.6	1.9	0.60
M*c* +	24.5	309.5	2.0	0.59
M*m*-	11.7	201.4	2.1	0.87
M*m* +	10.3	159.1	2.4	0.79
M*f*-	18.6	62.8	2.4	1.51
M*f* +	16.5	58.0	2.0	1.80

*D*_*50*_ median particle diameter, *span* (D_90_-D_10_)/D_50_, *Ssp* specific surface area

The chemical composition of each fraction was assessed by focusing on the analysis of the main cell wall components (polysaccharides, lignins, ester-linked *p*-coumaric acid and ferulic acid) that are known to be involved in enzymatic sensitivity (Table 2). If the total amount of sugars remained relatively stable between the fractions (about 66.7% ± 1.2%), the relative proportion of neutral sugars associated with hemicellulose (arabinose, xylose) was much more variable (relative standard deviation ranged from 8–15%). No major differences in glucose concentrations were observed. Cellulose crystallinity, determined in CP-MAS solid-state NMR experiments, was around 25% and no significant difference between fractions was observed. This CrI value is within the range of values determined by the NMR method for corn stover reported by [66]. Lignin content, measured using the Klason method, varied significantly from 14.52 to 19.56% in the fractions of cell wall material. Lignin structure was studied by thioacidolysis that estimates the monomer amount of guaiacyl (G), syringyl (S) and *p*-hydroxyphenyl (H) involved in β-O-4 bonds. The thioacidolysis yield ranged from 720 to 999 µmol.g^−1^ KL, suggesting different degrees of lignin condensation in the maize fractions. The S/G ratio of thioacidolysis monomers ranged from 0.9 to 1.42.

**Table 2:** Chemical composition of maize shoot powder and its fractions

Sample	Sugars (% dm)						Ash	Proteins	Acetyl	Lignin			CA	FA
	Total	Ara	Xyl	Glc	Gal	Uronic acids	(%dm)	(% dm)	(% dm)	Klason Lignin (% dm)	Thio yield (µmol.g^−1^ KL)	S/G	(mg.g^−1^ dm)	(mg.g^−1^ dm)
M	66.3	2.8	20.6	38.1	1.0	3.4	3.58	3.48	4.74	18.50	777.5	1.04	17.24	6.19
M*c*-	67.2	2.7	22.1	38.0	0.8	3.2	2.61	2.17	3.75	19.01	823.5	0.92	19.00	6.79
M*c* +	68.2	2.5	22.4	39.0	0.8	3.2	2.10	2.20	5.05	19.56	999.0	1.00	20.41	6.79
M*m*-	67.6	3.4	22.3	37.1	1.1	3.3	4.01	2.42	3.98	14.79	748.5	0.90	12.54	5.19
M*m* +	65.7	2.6	20.1	38.4	0.9	3.2	2.42	4.41	4.25	16.58	828.5	1.08	15.87	6.03
M*f*-	67.3	3.6	19.8	37.6	1.4	4.3	3.38	3.70	4.06	15.43	720.5	1.28	15.37	5.70
M*f* +	64.7	2.9	17.9	37.9	1.1	4.3	1.85	4.56	4.84	14.52	918.5	1.42	20.28	6.49

*Ara* arabinose, *Xyl* xylose, *Glc* glucose, *Gal *galactose, Klason lignin: expressed according to the dry matter of the extractive free material, Thio yield: expressed according to the amount of klason lignin (KL), *CA* ester-linked *p*-coumaric acid, *FA* ester-linked ferulic acid, *dm* dry matter

Major differences in composition were observed depending on the particle size of the fractions and opposed fine and coarse fractions. The coarse fractions, which could be seen as the most difficult to grind, showed higher concentrations of xylose and Klason lignin and also differences in lignin structure as evidenced by the lower S/G ratio. Lower concentrations of protein, galactose and uronic acid were also observed in the coarse fractions compared to the fine ones. Differences according to the electrostatic separation were also observed: the positively deviated samples (‘ + ’) had more acetyl groups, ester-linked *p*-coumaric and ferulic acid, and higher thioacidolysis yields, but lower ash content. Smaller differences were observed between the two coarse fractions compared to between the fine and medium fractions, in particular ash and phenolic acid contents. Electrostatic separation, which is based on surface properties, might be less efficient for bigger particles whose specific surface area is lower [59].

Likewise, particle size fractions isolated from the maize shoot powder differed in chemical composition, supporting the hypothesis that they are enriched in specific tissues. The higher concentrations of galactose and uronic acids in the finest fractions, associated with lower lignin content, suggest enrichment in pith from the stem [60, 61]. However, the higher amount of ash, also expected in this case [67], was not observed in this sample set. Leaves were also expected to be more present in the finest fractions [63]. In Poaceae, compared to the stem, leaves are characterized by less xylose and more protein, like in sorghum [68], more ash [69, 70], a lower S/G ratio, like in sugarcane, and less ester-linked *p*-coumaric [70]. More leaves in the fine fraction can be assumed from lower xylose or higher protein amounts. The fine fractions were found to be the richest in lignin syringyl units (%S > 55%), despite their lower lignin content. This observation suggests that these fractions are enriched in leaf blade sclerenchyma, a tissue repeatedly shown to contain lignins rich in S units [50, 71]. In negatively deviated fractions (‘−‘), an enrichment in ash was observed combined with a brownish hue (Fig. 2). This could reflect more leaves, even if protein enrichment was not that high. Nevertheless, based on our compositional analysis, we propose the following interpretation: coarse fractions could originate from the stem rind, while the fine fractions could be enriched in pith and leaves. The enrichment in leaves may also differ according to the electrostatic separation, with more leaves in the M*f*- fraction. Medium fractions could contain tissues from both origins, again with more leaves in the M*m*- fraction.

### Degradation kinetics by imaging and chemical analyses: Cinetore experiments

The parallel release of sugar and changes in the number and size of the particles during saccharification were analyzed in Cinetore experiments. The coarse fraction considered corresponded to the whole M*c* fraction (obtained before electrostatic separation) as the compositions of the two fractions M*c- and* M*c* + were close.

#### Fractions at time T0

**Fig. 3:**
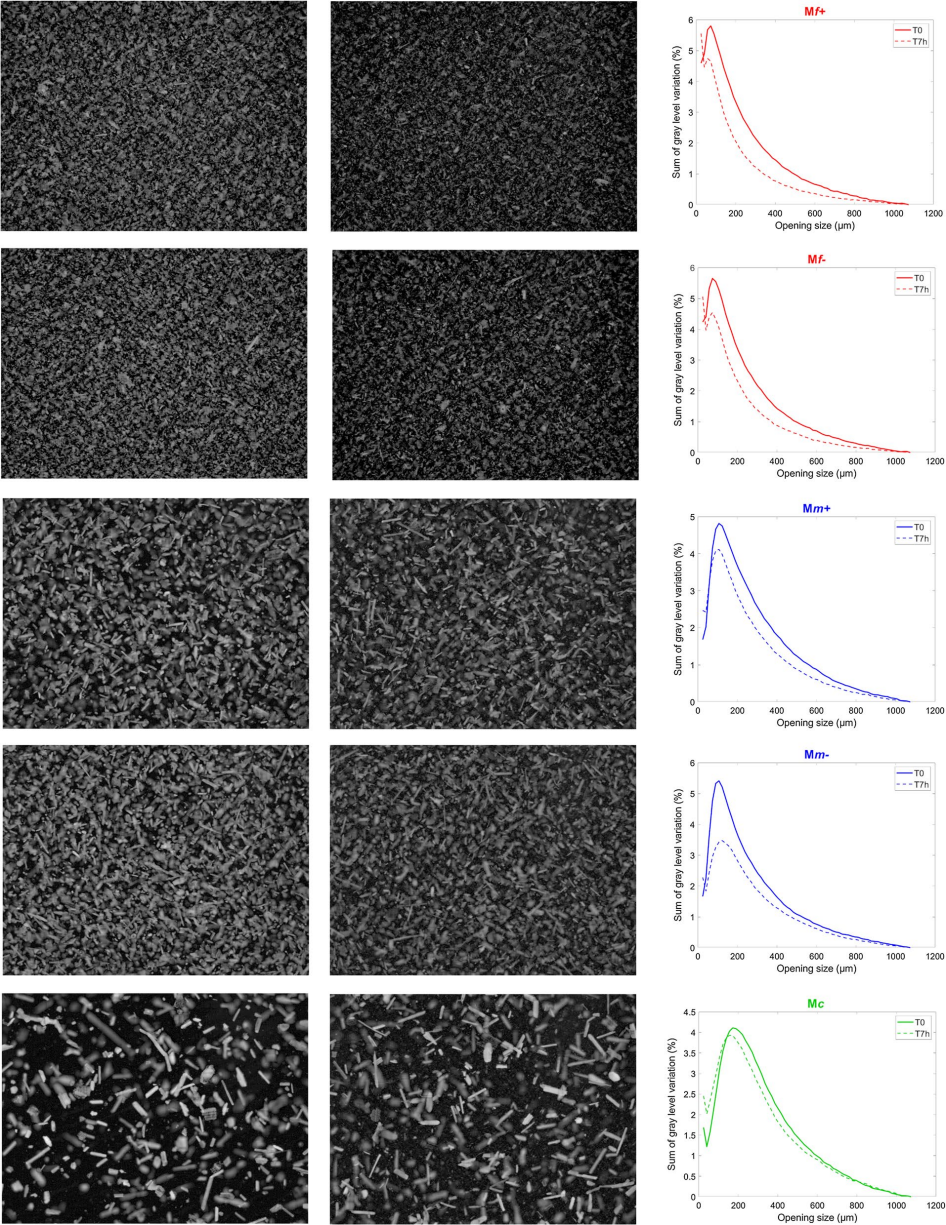
Cinetore analysis. Examples of image at times 0 and 7 h for maize shoot fractions: from top to bottom: M*f* + , M*f*-, M*m* + , M*m*- and M*c*. Field of view: 10.0 × 13.3 mm^2^. Brightness and contrast were set at 60 and -40 for the fine fractions and to 40 and -40, respectively, for the three other fractions. Granulometric curves of the original sample and after 7 h saccharification. T0: time T0 normalized to 1, T7h: time 7 h normalized relative to the number of particles at time T0

Examples of images at time T0 are given in Fig. 3 for the five fractions. Although the same mass of particles was loaded in the reactor, the images of particles differed considerably. At T0, particles in M*c* were mainly elongated with variable thickness and length. As expected, particles in the medium fractions (M*m* + or M*m*-) were clearly more numerous and differed in shape: thin and elongated to almost isotropic and cuboid shapes. In the raw images (see Additional file 1), particles in the fine fractions (M*f* + or M*f*-) were so numerous that they could hardly be distinguished. Considering the D_50_ measured by laser granulometry (60 µm) and the pixel size (8.2 µm), only a few pixels are required to observe one particle and their overlapping led to a low contrast between background and particles. The bright background attested to the high density of particles and to particles smaller than the pixel size. After subtracting the background (Fig. 3), many isotropic particles were observed together with small pointed particles and a few elongated particles. For these fine fractions, the estimation of the relative number of particles from the total gray levels would be underestimated and should be considered relative to the number of particles larger than 8.2 µm.

For the fine fraction, the mode was found at 55–75 µm while it was 90–125 µm and 155–205 µm for the medium and coarse fractions, respectively. The mode corresponded to the D_50_ (Table 1) in the case of the fine fractions and was lower for the other fractions. It should be noted that particle size evaluated by image analysis is more sensitive to the smaller dimensions of the particles [72], i.e. the width rather than the length, compared to laser granulometry. In addition, the variations in gray level observed for large sizes in the case of fine fractions were caused by the overlapping of the numerous particles. To a lesser extent, variations in the gray level were observed for small sizes in the medium and coarse particles, which actually corresponded to irregularities in their contour.

#### Changes in the number and size of particles during saccharification

After seven hours of saccharification (Fig. 3), particles could still be seen in the images, regardless of the fraction concerned. Fewer particles were observed in the coarse fraction M*c*, and the contrast was lower. In the fine fractions, the contrast was lower after seven hours compared to T0 and fewer particles could be distinguished. A decrease in the number of particles was also visible in the medium fractions. Considering the gray level granulometric curves normalized with respect to time T0, the lower intensity of the curves clearly confirmed the decrease in the number of particles in all the fractions, with the biggest decrease in the fine (M*f*-, M*f* +) and M*m*- fractions. In the fine fractions, a decrease in particle size was evidenced with an absolute increase in the number of particles smaller than 25 µm, whereas a moderate decrease in particle size was observed in the coarse and medium fractions.

**Fig. 4:**
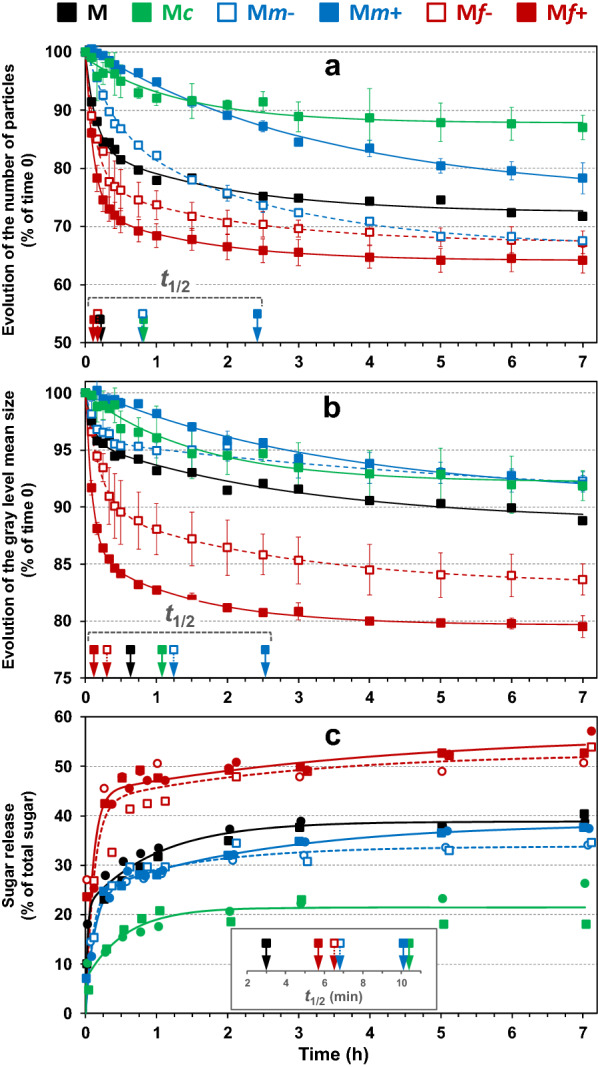
Kinetics of saccharification of maize shoot fractions (M*c*, M*m* + , M*m*-, M*f* + and M*f*-), in the reactor CINETORE. **a** Changes in the number of particles, **b** Changes in the mean particle size, **c** Sugar release (% of total sugars). For the evolution of particle size and quantity, the curves resulted from the two repetitions were averaged (mean values with standard error). For the sugar release, the two repetitions for each fraction were shown and differentiated by their symbols in the graph. The kinetics for the unfractionated maize shoot sample (M) were also plotted to highlight differences of saccharification after fractionation

To investigate physical changes in the particles depending on the period of saccharification in more detail, the number of particles was deduced from the total amount of gray levels, and mean particle size was estimated from the gray level mean sizes (Fig. 4a, b, respectively). The results obtained for the unfractionated maize shoot sample were plotted together with those of the fractions to highlight the saccharification differences after fractionation. In all the fractions except M*c*, the decrease in the number of particles was always greater than the decrease in particle size. The kinetics were analyzed through the time needed to obtain a 50% reduction in the number of particles or in their size (*t*_1/2_): in most cases the reduction in the number of particles took place more quickly than the reduction in particle size.

The least change in the number and size of particles was observed in the coarse fraction M*c*. The total decrease in gray levels between images after seven hours of degradation was 12% and the gray level mean size decreased by 8%. The size and number of particles decreased with quite similar *t*_1/2_ (*t*_1/2 _= 1.08 h and 0.82 h, respectively).

Conversely, the fine fractions (M*f* + and M*f*-) were the most impacted during saccharification: after 7 h, the total decrease of gray levels was 36% and 32% in M*f* + and M*f*, respectively, and the gray level mean size also decreased by 20% and 17% in M*f* + and M*f*-, respectively. For the sake of comparison, the decrease in the total gray level in the control experiments was 28% and 18% in M*f* + and M*f*-, respectively. Particle disappearance was clearly enhanced by enzymatic hydrolysis. The size and number of particles varied consistently with a rapid and important decrease occurring in the first 15 min. These two phenomena were observed following similar kinetics in the M*f* + fraction, whereas in the M*f*- fraction, the decrease in particle size was slightly slower (*t*_1/2_ = 19 min) than the decrease in the number of particles (*t*_1/2_ = 10 min).

Different patterns were observed in the medium fractions. In M*m*- samples, after seven hours of saccharification, almost no significant change in particle size (9%) was observed, while the number of particles decreased by about 34%. Mechanical stirring alone could not explain such a discrepancy as the gray level decrease in control experiments was only 4%. This result suggests that saccharified particles were fully dissolved in the aqueous medium with no noticeable particle erosion or breaking. The reduction in the total number of particles was similar to that obtained in the fine fractions but was less rapid (*t*_1/2 _= 49 min for M*m*- compared to *t*_1/2_ = 10 min and *t*_1/2_ = 7 min for M*f-* and M*f* + , respectively). This could suggest similar tissue composition but differences in surface accessibility (in relation to particle size) between M*m*- and fine particles. In the M*m* + sample, the particle size reduction was also very small (10%) but the reduction in the number of particles was also smaller (20–24%). Particle changes in M*m* + were the slowest observed in these fractions, as indicated by the higher values of *t*_1/2_ (around 2.5 h). The reduction in both the number and size of the particles was moderate, even less than in the coarse fractions during the first 90 min.

Differences in the physical change pattern were next observed according to particle size. Fine particles were the most impacted, regardless of the criteria used (number of particles or size), the coarse ones the least. Concerning the disappearance of particles, the medium fractions were between the two but concerning the decrease in particle size, their behavior was closer to that of the coarse fraction.

#### Sugar release during saccharification.

After seven hours of hydrolysis, sugar release was 22%, 34%, 38%, 52% and 55% in M*c*, M*m-*, M*m* + , M*f-*and M*f* + , respectively (Fig. 4c). The sugar release for the unfractionated maize shoot sample (M) was plotted together with those of the fractions to highlight the saccharification differences after fractionation. The main differences were observed according to the overall particle size of the fraction, with coarse, medium, and fine fractions ranked according to increasing hydrolysis yield. Concerning electrostatic separation, the medium and fine fractions obtained at the positive electrode were slightly more degraded than those recovered at the negative electrode.

Concerning kinetics, the saccharification of fine fractions was the most rapid as shown by the lowest *t*_1/2_ (around 6 min), while the coarse fraction had the highest *t*_1/2_ value (10.4 min). It could be noticed that the saccharification of the unfractionated maize stover was also rapid. As suggested by Mansfield et al. [73], the smallest size fractions were hydrolyzed preferentially during the first stage of the hydrolysis reaction. Contrasted kinetic values were observed for medium fractions: saccharification of M*m* + was as slow as in the coarse fraction (*t*_1/2_ = 10.1 min) whereas M*m*- behaved like the fine fraction M*f*-.

#### Coupled physical and chemical changes during saccharification

**Fig. 5:**
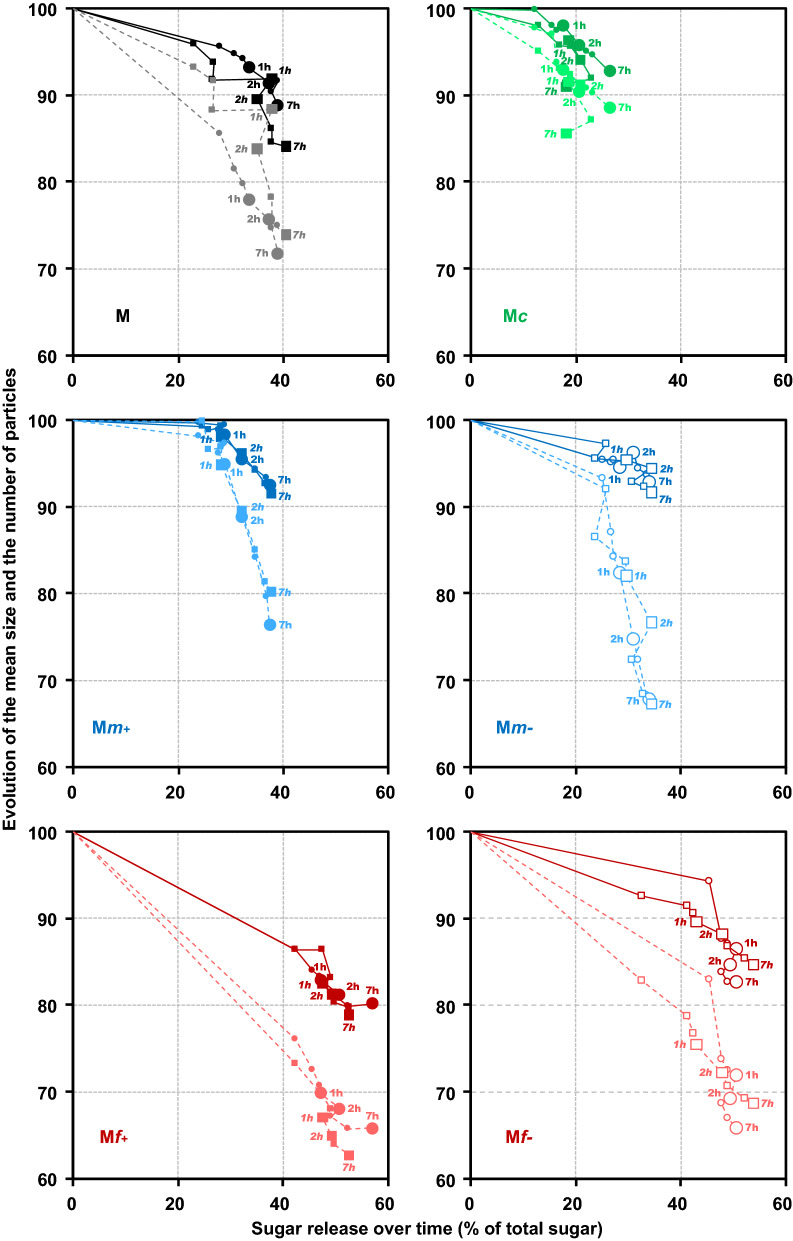
Saccharification of maize shoot fractions in the reactor CINETORE. Changes in the relative mean particle size (solid line) and relative number of particles (dotted lines) according to the relative percentage of neutral sugars released through enzymatic hydrolysis in fractions M, M*c*, M*m* + , M*m*-, M*f* + and M*f*-. Each point corresponds to different hydrolysis times (15 min, 30 min, 45 min 1 h, 2 h, 3 h, 5 h, 7 h

Saccharification was described by combining the analysis of saccharification yield, the reduction in the number and size of particles (Fig. 5). Whatever the fraction, the maximum relative change in the number of particles (dotted lines) was still lower than the chemical saccharification yields obtained at the plateau. The times to reach 50% of the modification were always shorter for chemical changes than for physical changes. This result suggests that at least some particles were fully degraded, as evidenced by the decrease in the total number of particles, while other particles were only partially degraded. Sugar release could be mainly due to the hydrolysis of few particles potentially originating from highly hydrolysable tissues, such as pith parenchyma [8] or leaves [74].

In general, the release of sugars was linked with a decrease in particle size (solid line in Fig. 5), but with different ratios. In the fine fractions, a high hydrolysis yield was observed with both a decrease in the number and size of the particles. The release of sugar and the decrease in both the number and size of the particles were very rapid in the fine fractions (M*f* + and M*f*-) as after 9 min, more than half of the degradation had already occurred. Conversely, in the coarse fraction M*c*, little change in either the number or the size of the particles was observed throughout saccharification. After one hour, both the size and the number of particles decreased slightly when a small extra quantity of sugar was released.

Medium fractions (M*m*- and M*m* +) showed distinct behaviors with no connection between chemical and physical changes. In M*m*-, some particle changes were observed together with the sugar release in the first stage of saccharification. After 1 hour of saccharification, the relative amount of particles continued to decrease whereas the saccharification yield had already levelled off. Physical changes continued to occur even if no more sugar was released, suggesting that partially hydrolyzed particles were broken down by stirring. In M*m* + , high sugar release (about 30% of the total sugars) was observed before 30 min, with no significant or only moderate change in both the number and size of the particles. After 30 min, the number of particles decreased dramatically whereas the increase in sugar release and the decrease in particle size were moderate. The delay in changes in the physical state relative to sugar release could be explained by the fact that sugars are easily accessible with no immediate visible modification of the particles. After a while, some particles are weakened by chemical degradation, and start to break down into small fragments and rapidly disappear. Indeed, the number of particles was more rapidly affected than their size. In this case, enzymatic hydrolysis did not have much effect on the morphology of the particles and may proceed from inside the particles.

### Relationships between physicochemical characteristics, water mobility distribution and saccharification patterns

**Fig. 6:**
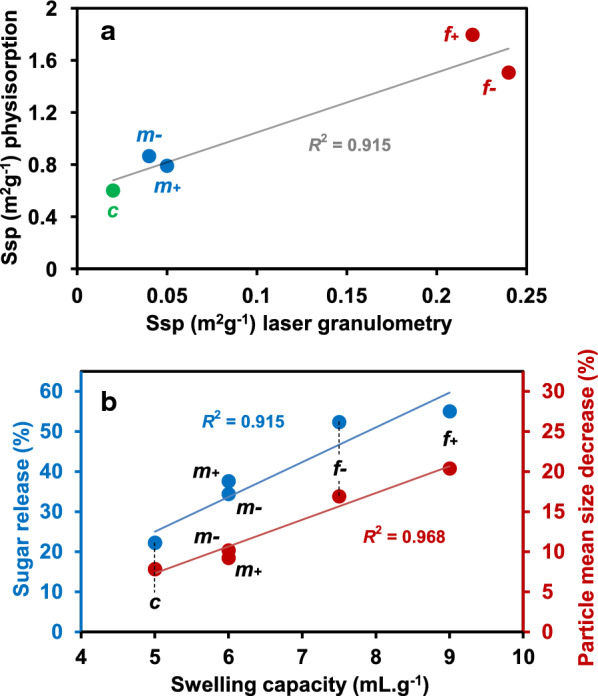
**a** Specific surface area of maize shoot fractions according to the method of evaluation used. **b** Inter-relationships between saccharification yield, particle mean size decrease, and swelling

High sugar release prior to the reduction in the number and size of the particles, observed particularly in the M*m* + fraction, could be explained by the open porosity of the particles. The specific surface (Ssp) area of all the fractions measured by physisorption was systematically higher than the Ssp area calculated from granulometric curves (Fig. 6a), also supporting the presence of open porosity at mesoscale (2–50 nm pore size). A high correlation (*R*^2^ = 0.915) was observed between the values obtained with the two methods. M*m* + did not deviate from this relationship (Fig. 6a). However, the specific surface area measured by physisorption corresponded to the surface area available to krypton molecules and but not to the surface accessible to cellulase, whose mean size is about 5.9 nm [75]. Moreover, values were determined in the dry state at -195.8 °C, quite far from hydrolysis conditions implying swollen substrates.

Swelling properties and water retention capacity (WRC) whose values include both water at the surface and between the particles and water within the biomass were considered (Table 3). The coarse fractions had the lowest values for both swelling and WRC, and the fine fractions the highest. A positive correlation was found between these properties (in particular swelling capacity) and sugar release (*R*^*2*^ = 0.915) and the reduction in particle size (*R*^*2*^ = 0.968) after seven hours of saccharification (Fig. 6b). However, here again, M*m* + did not differ from M*m*- and their values were intermediate between those of the coarse and the fine fractions (Table 3). Differences in swelling and WRC did not reflect the specific saccharification pattern of the M*m* + fraction.

**Table 3:** Water interaction properties of maize shoot powder and its fractions

Sample	Swelling capacity (mL.g^−1^)	Water retention capacity (g.g^−1^)
M	7.5	8.25
M*c*-	5.0	5.60
M*c* +	5.0	5.55
M*m*-	6.0	7.15
M*m* +	6.0	6.90
M*f*-	7.5	8.85
M*f* +	9.0	8.00

Low-field nuclear magnetic resonance (LF-NMR) was used to obtain information on water-biomass interactions at the molecular-scale. ${T}_{2}$ relaxation times of water proton distribution and their ${P}_{2}$ relative proportions were determined. To take advantage of the high sensitivity of this approach to any changes in sample supramolecular structure associated with water distribution and diffusion specificities, water-fraction interactions were investigated at five different moisture contents (MC) ranging from 15 to 67%. It should also be noted that at low water content (15% MC), the NMR signal is expected to be dominated by water arising from the hydration shell of macro-molecules and from water in interaction within the small pores of matrices, reporting on their microstructural specificities.

**Fig. 7:**
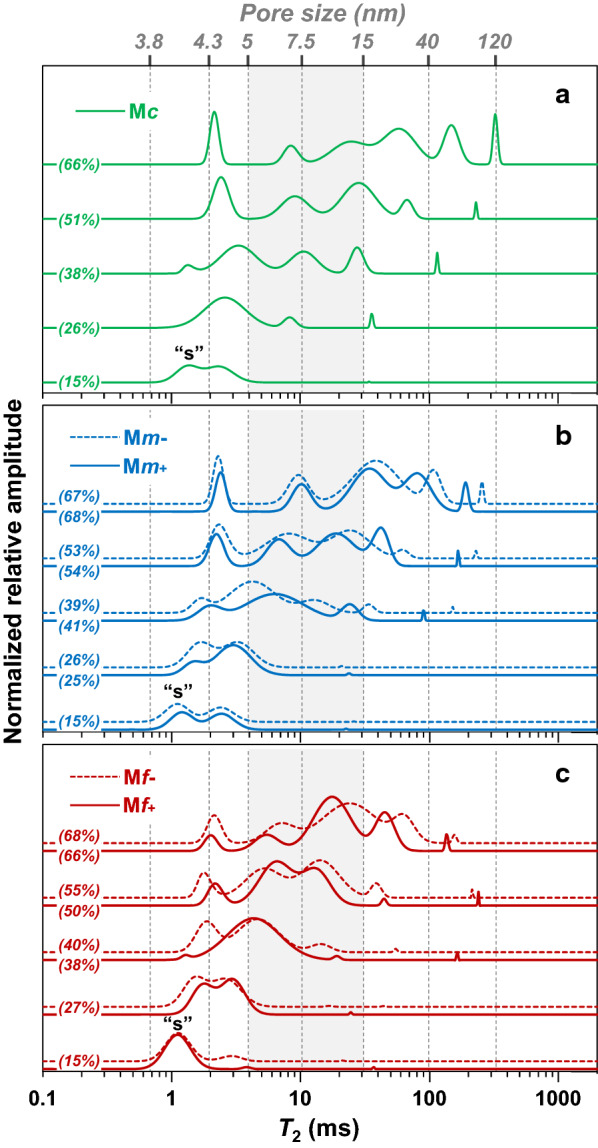
LF-NMR ${T}_{2}$ distributions for coarse (**a**), medium (**b**) and fine (**c**) fractions with increasing water content. The x-axis is logarithmic scale, and distribution amplitude is expressed in normalized relative water content (% w/w). The letter “s” denotes the shortest ${T}_{2}$ component (${T}_{2s}$) at 15% MC. The correspondence (vertical dashed lines) between mesoporosity (in nm) and ${T}_{2}$ relaxation times is indicated at the top of the Figure. The shaded area, between 5 and 15 nm (associated with ${T}_{2}$ values centered around 4 ms and 30 ms, respectively), indicate the range of pore sizes available for enzyme accessibility

As illustrated in Fig. 7, the analysis of ${T}_{2}$ relaxation curves led to ${T}_{2}$ profiles with multiple distinct peaks. Because no simple direct relationship exists between ${T}_{2}$ components and the morphological compartments in biological tissues [76], each ${T}_{2}$ peak can be preferentially assigned to a pool of water at a given range of mobility corresponding to specific molecular environment/interactions. Both the number of components, the relative surface area, and the individual mean ${T}_{2}$ relaxation time values associated with these peaks changed with increasing water concentration and differed between the five fractions M*c*, M*m*-, M*m* + , M*f*- and M*f* + . Overall, increasing moisture content led to an increase in the number of ${T}_{2}$ components (from 1–2 at 15% MC to 5–6 at 67% MC) with, in most cases, a shift towards higher water mobility modes, which could partially result from swelling. However, it should be noted that a short ${T}_{2}$ mode, centered around 2 ms, was present from 40 to 67% MC with only small changes in value/mobility, indicating that the physical–chemical environment associated with the high constraint water molecules can remain relatively unaffected in this range of moisture contents. In any case, differences between ${T}_{2}$ profiles of maize fractions were observed (Fig. 7), regardless of the particle size, the electrostatic deviation, and the water content.

**Fig. 8:**
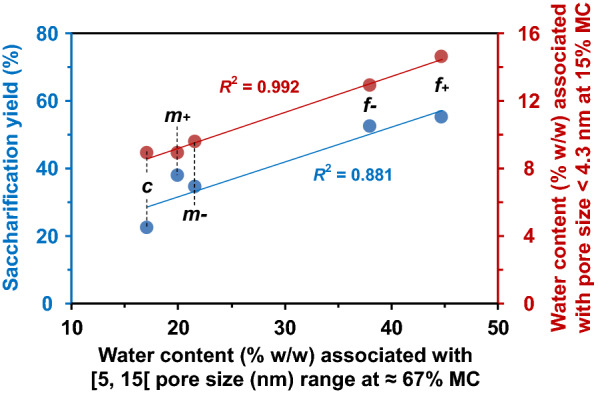
Inter-relationships between saccharification yield and water content associated with [5, 15[ pore size range (in nm), relative to the shaded area in Fig. 8, at 15% and 67% MC

**Fig. 9:**
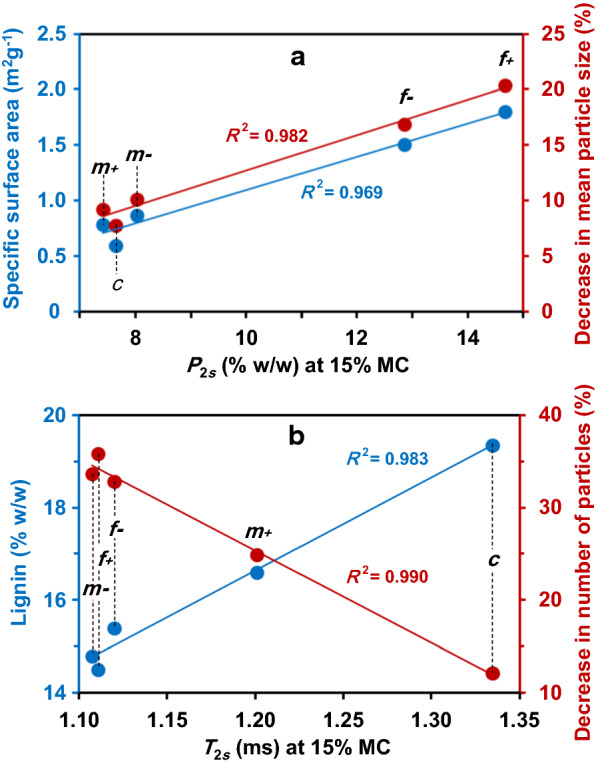
Inter-relationships between specific physicochemical characteristics. **a** Lignin content and reduction in the number of particles; **b** Ssp (specific surface area) and reduction in particle mean size and the NMR relaxation parameters associated with the shortest ${T}_{2}$ component “s”: **a** ${T}_{2s}$ and **b** ${P}_{2s}$

The water content associated with pores whose diameter are in the range [5. 15[ nm that typically represents the average diameter of enzymes, was investigated in more detail (Fig. 7, shaded area). The water content associated with this pore size range at 67% MC was positively correlated with the saccharification yield (*R*^2^ = 0.881) (Fig. 8). It was also correlated with the water content associated with pore sizes below 4.3 nm at 15% MC (*R*^2^ = 0.992). At 15% MC, the ${T}_{2}$ profiles of the five fractions showed two peaks with short relaxation times (≤ 4.3 ms). The population ${P}_{2s}$, associated with the ${T}_{2s}$ component, was positively correlated with specific surface area (Fig. 9a, *R*^2^ = 0.969), suggesting that this component is mainly influenced by water molecules located at the surface of particles. The ${T}_{2s}$ of M*m*- fraction was of the same order as that of the fine fractions, whereas the value measured for M*m* + was between the fine and coarse ones. Therefore, M*m-* differed from M*m* + by more constrained water, which is hypothesized to be essentially located at the surface of particles. Despite the very small change in the shortest relaxation time ${T}_{2s}$ value, it proved to be positively correlated with lignin content (Fig. 9b, *R*^2^ = 0.983). This could mean that the hydrophobic character of lignin may induce an increase in water mobility, in turn resulting in an increase in the relaxation time ${T}_{2s}$ centered from around 1.10 ms to 1.35 ms. The M*m* + fraction, with its higher lignin content, could keep the same macroscopic structure while still allowing accessibility to sugar hydrolysis.

Even at low moisture content, water/fraction interactions could be related to sugar release. The decrease in the number of particles was negatively correlated with the shortest ${T}_{2s}$ centered around 1.2 ms observed at 15% MC (Fig. 9b; *R*^2^ = 0.990) and the lignin content (*R*^2^ = 0.983). Moreover, the population ${P}_{2s}$, associated with the ${T}_{2s}$ component, was positively correlated with the reduction in mean particle size (Fig. 9a; *R*^2^ = 0.982). Thus, these two relaxometric parameters (${T}_{2s}$ and ${P}_{2s}$) could be early (at low MC) indicators of the saccharification potential, particularly when no significant swelling of the biomass matrix has yet occurred.

## Conclusions

Dry fractionation of plant biomass based on particle density and electrostatic properties has produced fractions of different chemical composition and physico-chemical properties, which may reflect variations in the relative proportions of tissue from either the stem or the leaves. Real-time monitoring of enzyme-induced sugar release and changes in the physical state of the particles clearly revealed different degradation patterns for these fractions. Depending on the fraction, changes in particle size and number were not necessarily synchronous with sugar release. Moreover, the relative decrease in particle size was much smaller than the relative decrease in particle number, suggesting (i) the coexistence of recalcitrant and degradable particles within a fraction: (ii) for degradable particles, chemical degradation within the particles with no visible change in size to a point where weakened, they break down and then degrade rapidly. Lignin was shown not necessarily hinder the access of enzymes to polysaccharides within the cell wall, but help to preserve the macroscopic structure of the particle despite high sugar release.

This paper also suggests that biorefinery processes could benefit from adding a dry fractionation step to optimise biomass flows and better exploit heterogeneity by diversifying uses according to fractions.

### Materials

The maize variety Maxxis grown at INRAE Lusignan center (France), was harvested at the silage stage. The maize shoot, i.e. without ears but containing both stems and leaves was oven dried at 55 °C and ground with a hammer mill with a 1-mm screen.

The whole ground maize shoot was separated into three fractions (coarse ‘*c*’, medium ‘*m*’ and fine ‘*f*’’) based on particle size using an air classification process (Hozokawa Alpine 50 ATP turboplex). The air classifier wheel was first set at 2,500 rpm rotor speed to collect the coarse fraction and the remaining fraction was then separated into medium and fine fractions with the rotor speed set at 4,000 rpm. Each fraction was further separated electrostatically (TEP System, Tribo Flow Separations, Lexington, U.S.A). Two fractions were recovered, one on the negative electrode side (coded “-”) containing the positively charged particles, one on the positive one (coded “ + ”), containing negatively charged particles. Particles in bulk were observed using the AZ100M Multizoom (Nikon, Japan) using white LED epi-illumination at low magnification (× 4).

The commercial preparations Celluclast® 1.5L, which is a cellulolytic complex produced by a selected strain of *Trichoderma reesei* cellulase, NS50010, which is a β-glucosidase (EC 3.2.1.21) and NS500030, which is an endoxylanase (EC 3.2.1.8) were gifts from Novozymes (A/D Bagsvaerd, Denmark). Prior to use, the three enzyme preparations were desalted on a PD-10 column (GE Healthcare Bio-Sciences AB, Uppsala, Sweden) to remove low molecular weight stabilizers. After desalting, the protein concentrations were 14.8 mg/ ml, 10.5 mg/ml. and 27.9 mg/ml for Celluclast® 1.5L, NS 50,010 and NS 50,030, respectively. The main activities of the desalted preparations were determined on model substrates (Table 4). Celluclast® 1.5L had a strong activity on carboxymethyl cellulose but also showed an activity on arabinoxylans. The NS50010 preparation has a strong β-glucosidase activity and NS50030 a strong activity on soluble and insoluble arabinoxylan and also on carboxymethyl cellulose. The three enzyme preparations were used in combination for the saccharification experiments and added in the proportions of 18, 24, and 17 mg of proteins/g of solid for Celluclast® 1.5L, NS 50,010 and NS 50,030, respectively.

**Table 4:** Specific activities of the three enzymatic preparations used for the saccharification tests

	De-salted enzymes	Celluclast® 1.5L	NS50010	NS 50,030
Polymerase activities (nkat/mg)	CMC*	98	5	35
	Soluble arabinoxylans	50	16	311
	Insoluble arabinoxylans	25	6	136
Glycosidase activities (nkat/mg)	pNP-Glc	7	124	4
	pNP-Xyl	11	1	11

Carboxymethyl cellulose (CMC), Nitrophenyl-β-d-Glucopyranoside (pNP-Glc) and 4-Nitrophenyl-β-d-Xylopyranoside (pNP-Xyl) were purchased from Sigma-Aldrich (St Louis MO)

Soluble and insoluble arabinoxylans were isolated from wheat flour in our laboratory

### Physical–chemical characterization

Except for particle size analysis, measurement of specific surface area and lignin composition, all the experiments were carried out on water-insoluble material. About 1 g of sample was suspended in 200 mL of distilled water containing 0.02% merthiolate (by weight). The suspension was stirred overnight at 40 °C and then filtered through a 10 µm nylon filter cloth. The insoluble fractions were washed with 50 mL of distilled water and then freeze dried.

#### Particle size analysis

The particle size distribution of the maize shoot fractions was measured using a Mastersizer 2000 laser diffraction particle size analyzer (Malvern Instruments Ltd., United Kingdom). Measurements were taken in ethanol, using 40% ultrasound power and setting obscuration at between 10 and 20%. Median diameter (D_50_), span ((D_90_-D_10_)/D_50_) and the calculated specific surface were extracted from particle size distribution expressed in volume from at least three parallel analyses per sample. The parameters D_10_, D_50_, and D_90_ indicate that respectively 10%, 50%, and 90% of the particles examined were below the corresponding diameter expressed in µm. The span represents the width of the particle size distribution.

#### Specific surface measurement

The Kr adsorption isotherms were determined using an ASAP 2460 (Micromeritics, France) at -195.8 °C. Samples (approx. 1 g) were first degassed under vacuum at 50 °C for 48 h (VacPrep 061, Micromeritics, France). The specific surface area was deduced from the Branauer-Emmett-Teller fitting method of isotherm at a partial pressure of between 0.02 and 0.3, and the mean relative deviation between duplicates was less than 3%. The measured surface area accounted for the whole rough external surface and for the surface accessible in the open porosity at the mesoscale (2 nm-50 nm pore size).

#### Swelling capacity

The swelling capacity of the powdered samples was measured using the bed volume technique [77]: 100 mg of sample was dispersed in 10 mL of distilled water in a graduated cylinder and left overnight at room temperature. The volume of the swollen sample was then measured and expressed as mL of swollen sample per g of original dry sample. The analysis was done in single.

#### Water retention capacity (WRC).

WRC was evaluated as the capacity of powder to hold water against a centrifuge force as described by Guillon et al. [78]. Three hundred mg of sample were suspended in 25 mL of deionized water and allowed to settle overnight at 4 °C under gentle stirring. The suspension was then centrifuged at 3,000*g* for 20 min at 20 °C and the supernatant was eliminated by passing the sample through a filter (mesh size 100 µm). The pellet was transferred to a weighed sinter (G2) to drain for 2 h. Sample fresh weight was recorded prior to drying at 103 °C overnight. The WRC was deduced from the water retained in the pellet divided by the sample dry mass. The mean relative deviation between duplicates was less than 4%. The analysis was done in duplicate.

#### Water mobility and pore size distributions

Water mobility and pore size distributions were measured by low-field nuclear magnetic resonance (LF-NMR) relaxation. Each fraction was left at controlled humidity (0.98 a_w_) for different times to reach the same moisture content (ranging from 15–67%).

LF-NMR analyses were carried out using a Minispec mq20 spectrometer (Bruker) operating at 0.47 T (20 MHz proton resonance frequency) equipped with a thermostated (± 0.1 °C) ^1^H probe. Fractions were packed in a 10-mm-diameter NMR tube to reach 1 cm in height and left for 10 min in the spectrometer for the temperature to stabilize at 4 °C. The transverse ${T}_{2}$ relaxation curves were acquired using a Carr–Purcell–Meiboom–Gill (CPMG) sequence. The 180° pulse separation was 0.2 ms, 1,024 even echoes were collected, and 2,048 scans were acquired with a recycle delay of 0.5 s resulting in a total acquisition time of about 20 min.

After acquisition, water was added to successively reach a hydration level of about 27%, 39%, 53% and 67%, and an interval of four days was left before the following measurements. The number of even echoes collected (2,048 to 20,000), the number of scans (1,024 to 128, respectively) and the recycle delay (from 1 to 5 s) were adjusted for each hydration state (from 27 to 67% w/w).

An inverse Laplace transformation (ILT) was applied to convert the relaxation signal into a continuous distribution of the ${T}_{2}$ relaxation components. For this purpose, a numerical optimization method was used by including non-negativity constraints and L1 regularization and by applying a convex optimization solver primal–dual interior method for convex objectives (PDCO) [79, 80]. Similar NMR acquisition and data treatment protocols were implemented on controlled pore glass samples of known diameters (Sigma Aldrich; pore size: 8, 25, 50 and 100 nm). A linear relationship (*R*^2^ = 0.998) between ${T}_{2}$ values and pore diameter was thus established and used to convert ${T}_{2}$ distributions of biomass fragments into pore size distribution. The analysis was done in single

#### Cellulose crystallinity

NMR experiments were performed on a Bruker AvanceIII-400 MHz spectrometer operating at a ^13^C frequency of 100.55 MHz and equipped with a double-resonance H/X CP-MAS 4-mm probe for CP-MAS solid-state experiments. Experiments were carried out at ambient temperature (20 C), in single for each experiment. Contact time was 1.5 ms. A typical number of 3,072 scans was acquired for each spectrum. Chemical shifts were calibrated with external glycine, assigning the carbonyl carbon at 176.03 ppm. To determine the degree of crystallinity of cellulose, the 78–90 ppm region of ^13^C spectra was decomposed as previously described [81] using Peakfit® software.

#### Chemical composition

Except for lignin measurements, all analyses were carried out on water-insoluble material. Lignin analyses were performed on extractive free material. Extractive-free material was prepared by exhaustive extraction with water and ethanol before drying at 40 °C. All the analyses were performed in duplicate except for uronic acid and ash content determined in a single test.

Neutral sugars were analyzed by gas chromatography (GC) after sample hydrolysis by sulfuric acid conversion of the monomers to alditol acetates as previously described [81, 82]. The mean relative deviation between duplicates was less than 5%. Total neutral sugars in enzymatic hydrolysates were quantified using the colorimetric orcinol method [83] with glucose as standard. The uronic acid content in the sulfuric acid and enzymatic hydrolysates was determined using the metahydroxydiphenyl colorimetric method [84] and glucuronic acid as standard. The constituent sugars were expressed as polysaccharides taking into account the loss of one water molecule for each glycosidic linkage. Therefore, experimentally determined values for monosaccharides were converted to polysaccharides using factors 0.88 for pentoses and 0.90 for hexoses. The total amount of proteins was estimated using the Kjeldahl method with a nitrogen protein conversion factor of 6.25 according to the standard NF EN ISO -5986–1 (2005/Cor;1:2008). The mean relative deviation between duplicates was 25%. Acetyl contents were measured by HPLC after alkaline hydrolysis according to [85] and the mean relative deviation between duplicates was less than 6%. Ash content was measured using the thermogravimetric method according to the standard NF EN ISO 2171 (2010).

Ester-linked *p*-coumaric and ferulic acids were measured after mild alkaline hydrolysis [86], with a mean relative deviation of 2% between duplicates.

Klason lignin content was measured according to Dence [87]. Lignin structure was investigated by thioacidolysis [88]. Lignin-derived monomers were analyzed via their trimethylsilyl derivatives by gas chromatography-mass spectrometry (GC–MS). The mean relative deviation between duplicates was less than 2%.

### Saccharification experiments

#### The torus reactor Cinetore

**Fig. 10:**
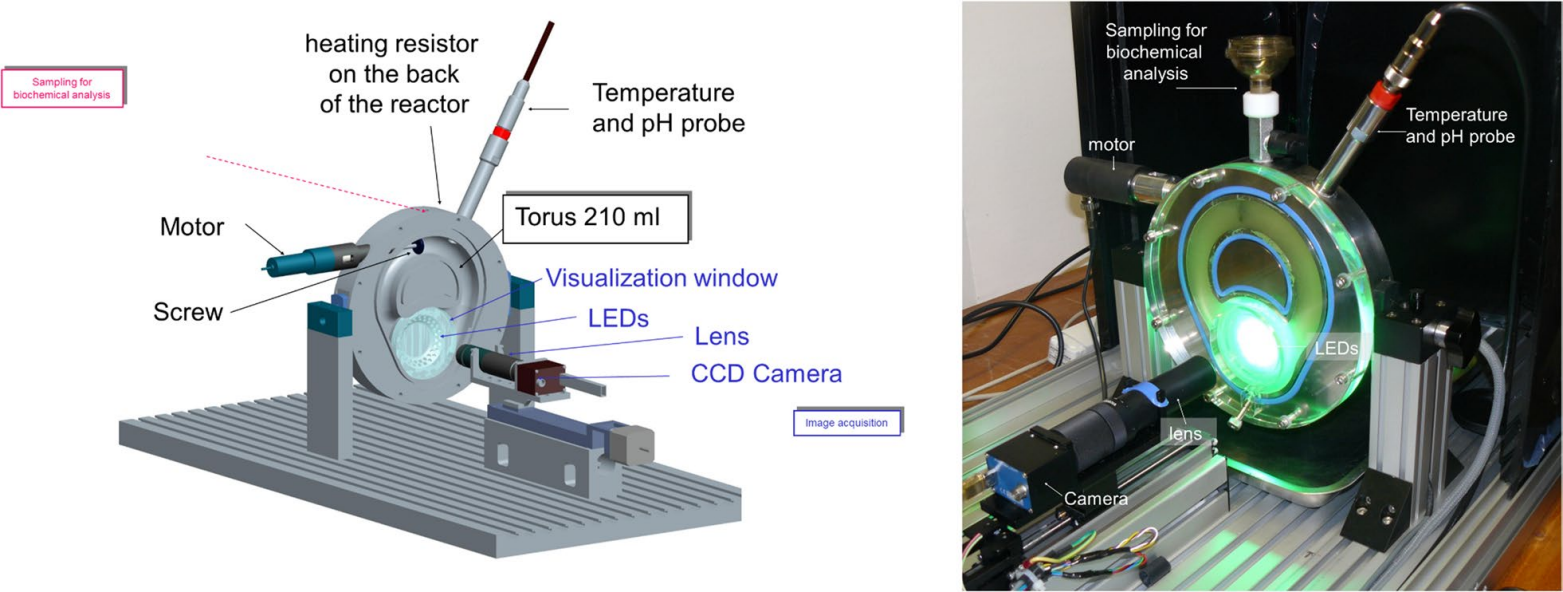
The torus reactor CINETORE. Scheme and photograph

Maize fractions were evaluated for saccharification kinetics in a torus reactor prototype named ‘Cinetore’ equipped for the visualization of particles at the macroscopic scale [89]. It is pictured in Fig. 10. The reactor is a steel block in which half a torus was machined. The diameter of the torus is 100 mm and the semi-circular section is 25 mm, giving a total volume of 205 mL. The geometry of the torus was distorted to allow the visualization of particles while maintaining a constant section volume. The reactor is closed by a transparent polycarbonate cover including a neutral video filter (*r* = 25 mm) as visualization window. The background of the steel block under the visualization window was painted black. Forty-eight green leds (525 nm Agilent HLMP-CM36-X10xx) were enclosed in the steel block around the visualization window at an angle of 30° to provide diffuse lighting of the samples through the particles. Particles, therefore, appear as bright objects against a black background. The Cinetore reactor was equipped with a monochrome camera (PROSILICA EC1600, Alliance Vision, Montélimar, France) that allows the acquisition of 1,220 × 1,620 pixels monochrome images with gray levels coded between 0 (black) and 255 (white). Flat resistances glued under the steel block and a thermocouple probe enabled the temperature inside the reactor to be controlled. Stirring is ensured by a three-blade screw driven by a variable speed motor. The reactor was placed in a vertical position with an opening at the top to allow the medium to be sampled for chemical analysis. A × 0.55 telecentric lens (T100/0.6 Vision Control, Alliance Vision, France) was used: the pixel size was 8.2 × 8.2 µm^2^, and the field of view 10.0 × 13.3 mm^2^.

#### Hydrolysis experiment

Four hundred mg of water-insoluble material was suspended in water and placed in the Cinetore reactor. The volume was completed to 200 mL with deionized water. Degradation took place at 40 °C for a period of seven hours in the presence of an enzymatic cocktail. The combination of enzymes (18, 24, and 17 mg of proteins/g of solid for Celluclast® 1.5L, NS 50,010 and NS 50,030, respectively) was added into the reactor. Experiments were performed in duplicate. In addition, one control experiment was performed with no enzyme.

The suspension was stirred throughout the reaction and the particles moved continuously inside the reactor. During the saccharification process, images of the particles were automatically acquired at 0, 5, 10, 15, 20, 25, 30 and 45 min, 1 h, 1 h 30, 2 h, 2 h 30 and 3, 4, 5, 6 and 7 h. Ten images were recorded on each occasion.

The chemical degradation of particles was evaluated by measuring the amount of sugars solubilized. Hydrolysates were sampled at times 0, 15, 30, 45 min, 1, 2, 3, 5, and 7 h. The hydrolysates were boiled for 10 min to inactivate the enzymes, centrifuged for 5 min (8,500 rpm, Sigma 6K10). Concentrations of uronic acid and neutral sugars in the supernatants were measured using colorimetric methods. The amount of sugar released at each measurement time is expressed as % of the total sugars in the original material (T0).

The kinetics of sugar release were modeled with an exponential law (one or two exponentials, depending on the fraction considered) to evaluate asymptotic values and, in particular, to extract the parameter *t*_1/2_, defined as the time necessary to obtain 50% of the full range of variation.

#### Image analysis

##### Image preprocessing

Examples of raw and preprocessed images are provided in Additional file 1 in which the particles appear as bright objects against a dark background. The general background of images changed during enzymatic degradation. Background variations were removed using the *tophat* transformation from mathematical morphology [72]. The *tophat* transformation selects bright objects smaller than a mask of a given shape and size called *structuring elements*. In the present work, a squared structuring element was considered with a side measuring 131 µm corresponding to 1,075 µm.

##### Number of particles

The total number of particles was evaluated as the sum of gray levels of preprocessed images at time T0. The values of the 10 images acquired at T0 were averaged to obtain the original value. The decrease in the number of particles during the reaction was measured as the percentage of relative decrease with respect to the original values. Again, values of 10 images acquired at each measurement time were averaged.

##### Particle size evolution

A huge number of particles was observed in the images making individual particle identification irrelevant (Fig. 1). Particle size evolution was therefore evaluated overall from the total particle population with no segmentation steps. Gray level granulometry based on mathematical morphology tools was used for this purpose [72]. It was applied as described by [89, 90]. The method provides granulometric curves that can be compared to usual granulometric distributions except that they are based on measurements of variations in the gray level. The technique does not extract the so called “actual size distribution” but enables comparison of images in a given experiment. Mean sizes can be calculated from the granulometric curves that were called “gray level mean size” [90].

In the present work, granulometric curves were computed using *morphological opening* by *linear structuring elements* [72] to account for the fact that many particles were elongated. Because of the random orientation of particles in the images, two orientations of the structuring elements (0 and 90°) were applied. Granulometric curves, therefore, measured the distribution of the average projected length of particles at 0 and 90°. The maximum size analyzed was 1,075 µm with a 16.4 µm step between two sizes. The 0 and 90° granulometric curves of the 10 images acquired at a given time were averaged to obtain a single granulometric curve per measurement time.

Granulometric curves are usually normalized with respect to the total gray levels of the image considered, i.e. the total number of particles in the image. In the present work, a second kind of normalization was used for the total gray levels of the image at time T0 for all images of a given kinetic, i.e. the total number of particles in the original image at time T0.

Gray level granulometric curves were computed using homemade software developed with Matlab and available at http://www.pfl-cepia.inra.fr/index.php?page=granulomorphogui_en).

## Supplementary Information

**Additional file 1**.

## Data Availability

The datasets used and/or analysed during the current study are available from the corresponding author on reasonable request.

## Abbreviations

D_50_: Median diameter of particle size distribution expressed in volume
FA: Ferulic acid
CA: Para-coumaric acid
Ssp: Specific surface area
WRC: Water retention capacity
MC: Moisture content (wet basis
