# Supplementary material for: Enzymatic degradation of maize shoots: monitoring of chemical and physical changes reveals different saccharification behaviors
Source: Biotechnol Biofuels. 2021 Jan 5;14:1. doi: 10.1186/s13068-020-01854-1 (PMC7786969; doi:10.1186/s13068-020-01854-1)

Image preprocessing


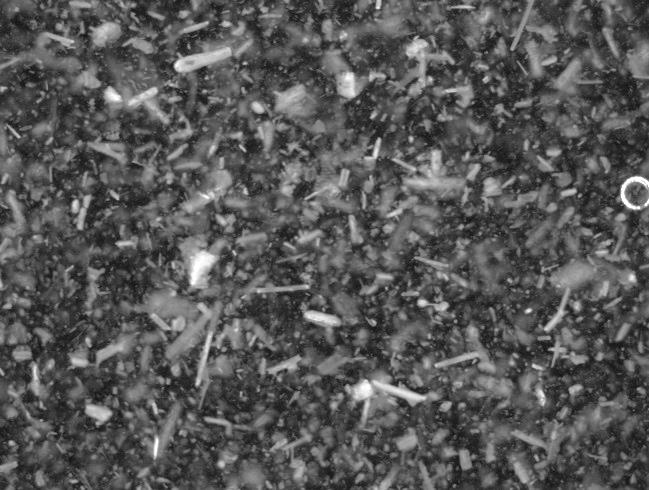

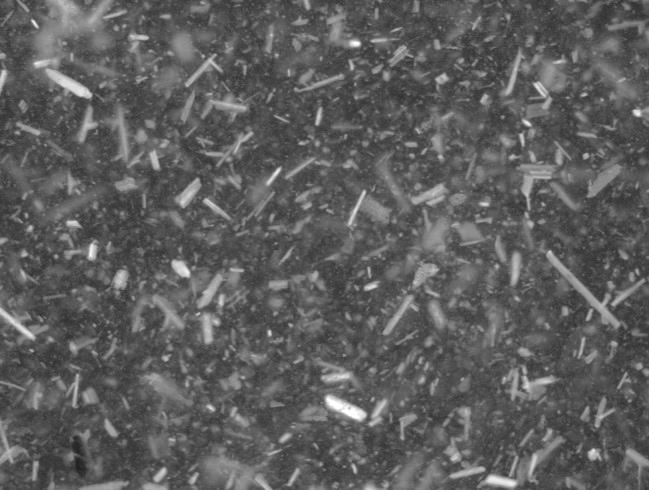


Examples of original images of maize shoot fractions at times 0 and 7 hours.


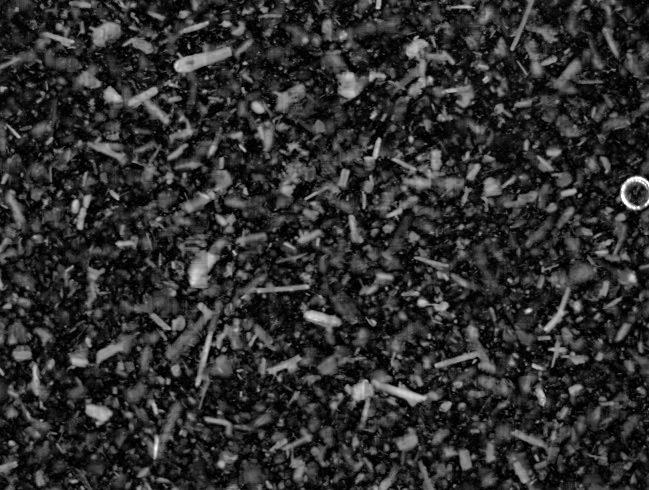

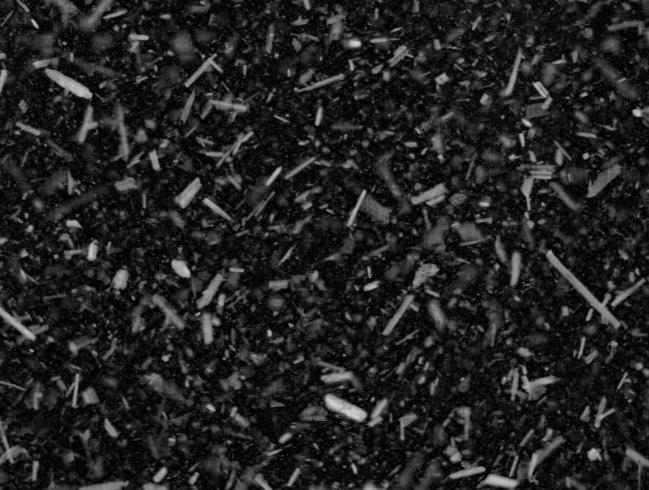


Images of particles after removing the background from original images. A decrease of total amount of particles is observed.

For all images, contrast and brightness were adjusted in a similar way and can be compared.

Analysis of Fractions. Examples of raw images at timeq 0 and 7h

M1f+
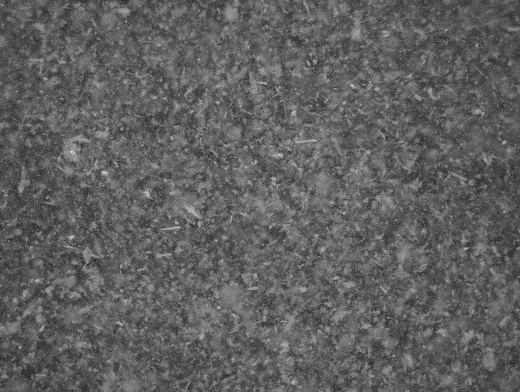

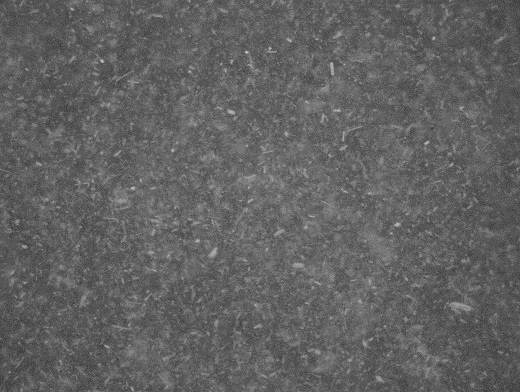


M1f-
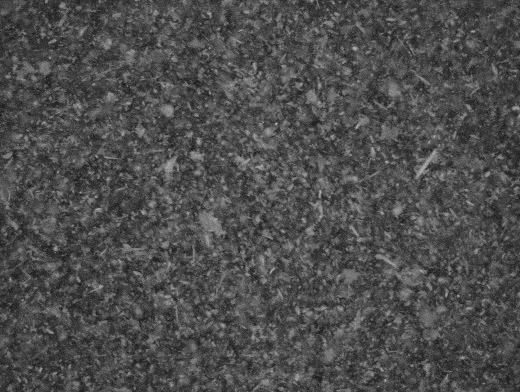

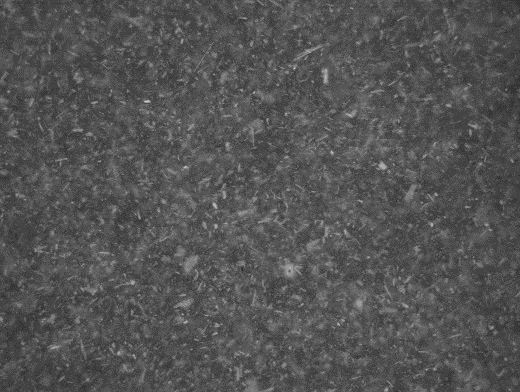


M1m+
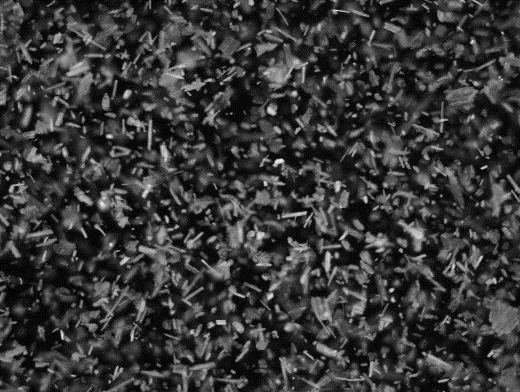

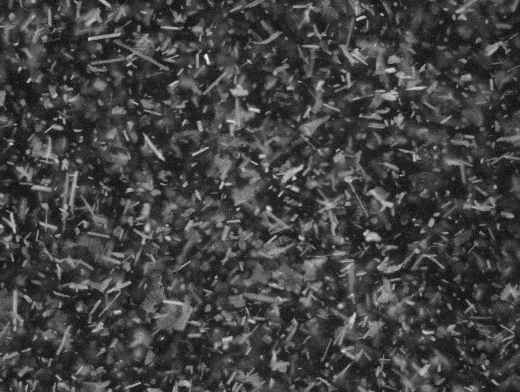


M1m-
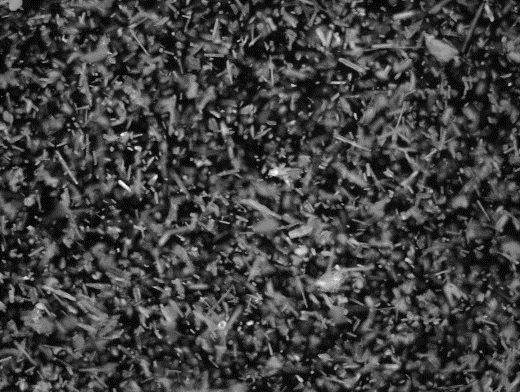

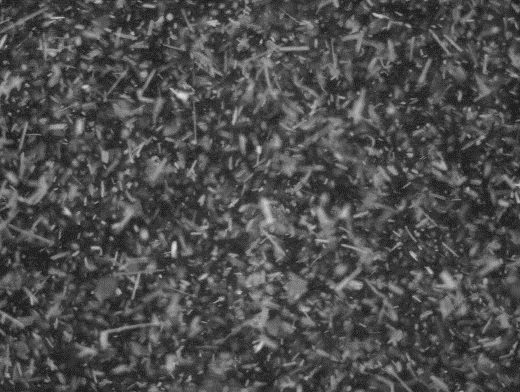


M1c
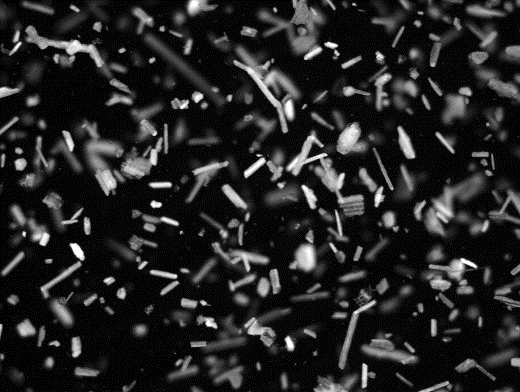

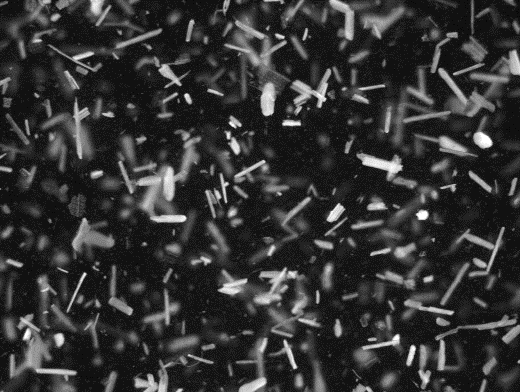

Supplement: Supplementary file 1 — Additional file 1. [file 13068_2020_1854_MOESM1_ESM.docx]
